# Matrix Metalloproteinase-9 as a Biomarker for Diagnosis and Monitor Disease Progression in Psoriatic Arthritis

**DOI:** 10.31138/mjr.151024.mpr

**Published:** 2025-07-09

**Authors:** Thomas Prisila, Ping Seung Ong, Mohd Shahrir Mohamed Said, Asrul Abdul Wahab

**Affiliations:** 1 Department of Medicine, Hospital Taiping, Jalan Taming Sari, Perak, Malaysia;; 2 Rheumatology Unit, Department of Medicine, Hospital Raja Permaisuri Bainun, Perak, Malaysia;; 3 Rheumatology Unit, Department of Medicine, UKMMC, Jalan Yaacob Latif Kuala Lumpur, Kuala Lumpur, Malaysia;; 4 Department of Medical Microbiology and Immunology, Department of Medicine, UKMMC, Jalan Yaacob Latif Kuala Lumpur, Kuala Lumpur, Malaysia

**Keywords:** psoriatic arthritis, matrix metalloproteinases 9 (MMP-9), disease activity, biomarker, diagnosis

## Abstract

**Objective:**

To evaluate the potential of Matrix Metalloproteinase-9 (MMP-9) as a diagnostic marker for psoriatic arthritis (PsA) and to investigate the correlation between MMP-9 levels and PsA disease activity.

**Patients and method:**

A total of 72 subjects participated in this cross-sectional study, consisting of 43 patients diagnosed with Psoriatic Arthritis (PsA) and 29 healthy control subjects. The Composite Psoriatic Disease Activity Index and Disease Activity in Psoriatic Arthritis were utilised to evaluate the disease activity levels in PsA patients. To measure serum levels of MMP-9 the quantitative sandwich enzyme-linked immunosorbent assay method was applied. Results: The mean age of PsA patients is 43.81 ± 12.82 years, with a mean BMI of 29.46 ± 5.91 kg/m^2^, significantly higher than healthy subjects (p < 0.01). Oneway ANOVA indicates a significant difference in serum MMP-9 levels among active PsA, inactive PsA, and healthy controls [F(2, 68) = 21.15, p < 0.001]. Serum MMP-9 levels significantly differ between PsA groups and healthy controls (p < 0.001). Pearson correlation shows no link between serum MMP-9 levels and PsA activity. MMP-9 shows strong diagnostic potential for distinguishing PsA patients from healthy controls, with an AUC of 0.88 (p < 0.001).

**Conclusions:**

This study demonstrated that MMP-9 shows promising potential as a diagnostic marker for PsA. but no significant correlation between serum MMP-9 levels and PsA disease activity. These findings highlight the need for further research involving a larger cohort of PsA patients to assess whether MMP-9 could play a complementary role in PsA diagnosis.

## INTRODUCTION

Psoriatic arthritis (PsA) is an inflammatory arthropathy associated with psoriasis, arthritis, enthesitis, spondylitis, and dactylitis.^1^ The Annual Report of the Malaysian Psoriasis Registry (2007–2019) reported psoriatic arthropathy in 13.8% of the adult psoriasis population.^2^ Wahinuddin et al. highlighted that the mean age of PsA patients in Malaysia was around 50 years, with an earlier onset of psoriatic arthritis observed among the Malay population, compared to the Chinese and Indian populations, which form the three major ethnic groups in Malaysia.^3^

Disease activity in PsA is typically assessed using composite measures, which combine various aspects of the disease into a total score to gauge the overall level of activity. Several tools are available to measure PsA disease activity, including Composite Psoriatic Disease Activity Index (CPDAI), Disease Activity in Psoriatic Arthritis (DAPSA), Psoriatic Arthritis Disease Activity Score (PASDAS), the Grappa Composite Score (GRACE) or Minimal Disease Activity (MDA).^4^ Each of these measures takes into account various manifestations of PsA, such as joint involvement, skin disease, enthesitis, dactylitis, and functional impact, providing a more comprehensive understanding of the disease’s activity level.

DAPSA provides a validated continuous score of arthritis activity, which has been shown to correlate with functional status and structural progression on radiographs, further supporting its validity. However, the use of C-reactive protein (CRP) in the DAPSA calculation has raised concerns, as CRP may not be an ideal inflammatory marker to assess PsA activity comprehensively.^5^ Although various disease activity measures are available and widely used in PsA research, there is no consensus on which measure should be adopted as the standard. The significant heterogeneity across the clinical domains of PsA presents challenges for accurate and consistent disease activity assessments. Given these complexities, there is a growing interest in developing biomarkers to help in the quantitative assessment of disease activity in PsA. These biomarkers could also play a crucial role in identifying patients at increased risk of developing PsA, improving early diagnosis, and guiding targeted therapeutic interventions.^6^ Matrix metalloproteinases (MMPs) are zinc-dependent enzymes that play a crucial role in the destruction of articular cartilage. These enzymes are particularly important in joint pathologies, including PsA. The expression of MMP genes is up-regulated in response to elevated levels of pro-inflammatory cytokines and soluble mediators such as tumour necrosis factor-α (TNF-α), interleukin-1 (IL-1), IL-6, IL-17, and interferon-γ (IFN-γ).^7^ These cytokines bind to their respective receptors on immune cells, macrophages, chondrocytes, synoviocytes, and osteocytes, initiating signalling cascades such as the mitogen-activated protein kinase (MAPK) and Janus kinase/signal transducers and activators of transcription (JAK/STAT) pathways. These pathways ultimately lead to increased synthesis of MMPs.^7^ Glazewska et al. highlighted the diagnostic specificity (DSp) of various MMPs and tissue inhibitors of metalloproteinases (TIMPs), finding that MMP-9 had the highest DSp (92.5%) for both the total psoriasis patient group and those with moderately active disease, making it a valuable biomarker in this context.^8^ In another study, Chandran et al. compared several soluble biomarkers to differentiate patients with PsA from those with psoriasis, concluding that MMP-3 is a significant biomarker for identifying PsA.^9^ These findings collectively underscore the importance of MMPs, especially MMP-9 and MMP-3, in the assessment and differentiation of inflammatory arthropathies like PsA.

This study aimed to evaluate the potential of MMP-9 as a diagnostic marker for PsA and to investigate the correlation between MMP-9 levels and PsA disease activity.

## PATIENTS AND METHOD

This is a case-control study conducted between June 2021 until March 2022 among patients attending rheumatology clinic in Pusat Perubatan Universiti Kebangsaan Malaysia (PPUKM) and Hospital Raja Permaisuri Bainun. All the patients diagnosed with PsA were examined by a rheumatologist assisted by candidate as the co-investigator and fulfil the CASPAR criteria.^10^ This study was approved by the PPUKM Research Ethics Committee (UKM PPI/111/8/JEP-2021-254) and National Medical Research Registry (NMRR) of Malaysia (NMRR 21-400-58804).

**Figure 1.**
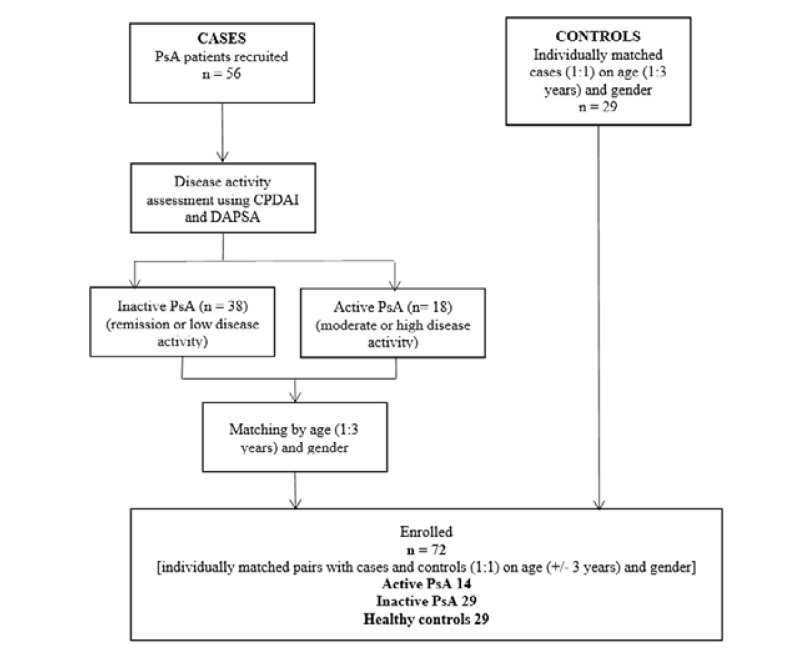
Flowchart of number of recruited patients in the study.

Eligible patients who fulfilled the inclusion and exclusion criteria had their demographic data and clinical parameters such as weight, height and body mass index (BMI) recorded. To ensure a balanced comparison, patients with PsA were matched with healthy controls based on age and gender. Following this matching process, the study included 14 participants with active PsA, 29 with inactive PsA, and 29 healthy controls. Patients in the PsA groups who did not match the age and gender criteria were excluded from this study. This ensured a more balanced comparison between groups and helped to reduce bias in the analysis. The distribution of participants across these groups is illustrated in **Figure 1**.

We excluded all patients with no clear diagnosis of psoriatic arthritis, history or active malignancy, concurrent with other autoimmune diseases, ischaemic heart disease, congestive heart failure, hepatitis B, hepatitis C, human immunodeficiency virus (HIV), diabetes mellitus with target organ damage, active inflammatory bowel disease, history of active tuberculosis, not consented for the study and pregnancy. Healthy controls were recruited from healthy volunteers who did not have psoriasis, inflammatory arthritis, acute illness, chronic illness or pregnant.

CPDAI and DAPSA were used to assess the disease activity of the PsA patient. CPDAI assess multiple domains of PsA including peripheral joints, skin, enthesitis, dactylitis, and axial disease to measure the disease activity and disease impact which are summed together to create total score of 0–15.^11^ DAPSA is an index to measure the disease activity of PsA. Thresholds 4,14,28 were used to differentiate between remission, low disease activity, minimal disease activity, and high disease activity.^12^ Patients who achieved remission or maintained low disease activity were categorised under the inactive PsA group.

Serum MMP-9 was collected into a sodium heparin tube and centrifuged for 15 min at 1000×g to obtain plasma samples and stored at –20°C until assayed. Serum concentrations of MMP-9 measured with the quantitative sandwich enzyme-linked immunosorbent assay (ELISA) according to the manufacturer’s instructions. The samples run in duplicate. The sample tests were run in triplicate if the coefficient variability (CV) is more than 10%. The mean optical density (OD) used for the measurement of the MMP-9 concentration.

Continuous descriptive data were expressed as mean and standard deviation for normally distributed variables, and for non-normally distributed data, median and interquartile range (IQR) were used. Descriptive data for categorical variables were expressed as numbers and percentages. To assess differences between the three groups (active PsA, inactive PsA, and healthy controls), a One-way Analysis of Variance (ANOVA) test was applied. Correlation between serum MMP-9 levels and PsA disease activity was evaluated using Pearson correlation. The diagnostic accuracy of MMP-9 markers in distinguishing PsA from healthy controls was determined using Receiver Operating Characteristic (ROC) curve analysis. A p-value <0.05 was considered statistically significant. All statistical analyses were performed using the Statistical Package for the Social Sciences (SPSS) for Windows, version 27.0 (SPSS Inc., Chicago, IL, USA).

## RESULTS

A total of 72 patients who met the inclusion and exclusion criteria were enrolled in this study, consisting of 43 PsA patients and 29 healthy controls. Among the PsA patients, 15 (34.9%) were male, and 28 (65.1%) were female. The majority of PsA patients were Malays, accounting for 25 (58.1%). The clinical manifestation of PsA in this cohort was predominantly distal interphalangeal joint involvement, seen in 19 patients (44.2%). There were only 2 patients with active psoriasis and both less than body surface area (BSA) 3%. Furthermore, 33 patients (76.7%) were being treated with conventional disease-modifying antirheumatic drugs (DMARDs) and 1 patient on biologic DMARDs. Regarding disease activity, the median score for the CPDAI was 4 [interquartile range (IQR) 3], while the median score for the DAPSA was 9.32 (IQR 11.45), indicating moderate disease activity (**Table 1**).

**Table 1.** Characteristics of psoriatic arthritis patients and healthy control group.

	**Total (n=72)**	**Total PsA (n=43)**	**Active PsA (n=14)**	**Inactive PsA (n=29)**	**Healthy Control (n=29)**	**P value**
Age (y)		43.81± 12.82	43.36 ±0.13	44.03±14.41	44.10±14.33	0.984
Gender						
Male	27(37.5)	15(34.9)	3 (21.4)	12(41.4)	12(41.4)	0.384
Female	45(62.5)	28 (65.1)	11(78.6)	17(58.6)	17(58.6)	
**Ethnicity**						
Malay	39(54.2)	25(58.1)	7(50)	18(62.1)	14(48.3)	0.225
Chinese	14(19.4)	8(18.6)	1(7.15)	7(24.1)	6 (20.7)	
Indian	18(25)	9(20.9)	6(42.9)	3(10.3)	9(31)	
Other	1(1.4)	1(2.3)	0	1 (3.4%)	0	
Duration of Pso(y)		14.40 ± 9.5	13.14±10.14	15 ± 9.30		0.363
Duration of PsA(y)		8.53± 8.52	9.07±9.25	8.28± 8.30		0.765
BMI (kg/m^2^)	27.48 ± 5.89	29.46±5.91	31.79±4.25	28.34±6.32	24.54±4.57	**<0.01**
**Co-morbidity**						
DM		9(20.9)	4 (28.6)	5(17.2)		0.392
Hypertension		16 (37.2)	5(35.7)	11(37.9)		0.888
Dyslipidaemia		13 (30.2)	2(14.3)	11(37.9)		0.114

Data presented as standard deviation with median below or as n (%).

PsA: psoriatic arthritis; Pso: psoriasis; y: year; DM: diabetes mellitus.

**Table 2** shows mean range for erythrocyte sedimentation rate (ESR), CRP and MMP-9 level comparing all the three groups. The median score for ESR is 13 (IQR 21) mm/hour, CRP is 1.27 (IQR 12.53) mg/L and mean range for serum MMP-9 level is 9.97 ± 5.71 ng/mL.

**Table 2.** Acute phase reactants (ESR, CRP) and serum MMP-9 level.

	**Total (n=72)**	**Active PsA (n=14)**	**Inactive PsA (n=29)**	**Healthy control (n=29)**	**P Value**
**ESR(mm/hour)^a^**	13(21)	2(1)	26(25)	12(12)	< 0.01
**CRP (mg/L) ^a^**	1.27(12.53)	27.5(33.75)	0.48(1.21)	1.10(3.6)	< 0.01
**MMP-9 (ng/mL)^b^**	9.97 ± 5.71	13.43 ± 3.99	12.56 ± 5.37	5.71 ± 3.82	< 0.01

a median (IQR) for non-parametric distributed data.

b mean ± SD for normally distributed data.

ESR: erythrocyte sedimentation rate; CRP: C-reactive protein; MMP-9: Matrix metalloproteinases-9.

For the serum MMP-9 level, there is significant differences between all the three groups. One-way ANOVA test shows statistically significant result, F(2, 68) = 21.15, p <0.001. A Tukey post hoc test revealed the comparison between the groups of active PsA with healthy control which shows statistically significant, 7.77, 95%CI, [4.19, 11.36] and inactive PsA with healthy control which shows statistically significant, 6.91, 95%CI, [4.00, 9.80]. However, there is no significant difference between active and inactive group (p = 0.829) (**Table 3**).

**Table 3.** Post hoc analysis comparing ESR, CRP, and MMP-9 with disease activity.

		**Healthy Control**	**Active PsA**	**Inactive PsA**
**MMP-9^a^**	9.97 ± 5.71			
**Healthy Control**	5.71 ± 3.82		<0.001*	<0.001*
**Active PsA**	13.43 ± 3.99	<0.001*		0.829
**Inactive PsA**	12.56 ± 5.37	<0.001*	0.829	

a mean ± SD for normally distributed data. bmedian (IQR) for non-parametric distributed data.

** significant level p<0.01;

* significant level p<0.05.

ESR: erythrocyte sedimentation rate; CRP: C-reactive protein; MMP-9: Matrix metalloproteinases-9.

Pearson correlation test used to assess correlation between PsA disease activity and serum MMP-9 level (**Table 4**). For CPDAI active arm with serum MMP- 9 level was found to be in negative correlation and statistically not significant (r = − 0.281, p = 0.331). As for DAPSA active arm with serum MMP-9 level found to very low positive and statistically not significant (r = 0.022, p = 0.939). For CPDAI inactive arm, serum MMP-9 level was found to be low positive correlation and statistically not significant (r = 0.038, p = 0.844). For DAPSA inactive arm, serum MMP-9 level found to positive and statistically not significant (r = 0.311, p value 0.1). From these findings, we can conclude that serum MMP-9 level is unable to correlate disease activity of PsA in this study.

**Table 4.** Correlation between MMP-9 with PsA Disease Activity.

**PsA active arm (n = 14)**				
**Variables**		**CPDAI**	**DAPSA**	**MMP-9**
**CPDAI**	Pearson correlation		0.848**	−0.281
	*p*-value		<0.001	0.331
**DAPSA**	Pearson correlation	0.848**		0.022
	*p*-value	<0.001		0.939
**MMP-9**	Pearson correlation	−0.281	0.022	
	*p*-value	0.331	0.939	
**PsA inactive arm (n = 29)**				
**Variables**		**CPDAI**	**DAPSA**	**MMP-9**
**CPDAI**	Pearson correlation		0.413*	0.038
	*p*-value		0.026	0.844
**DAPSA**	Pearson correlation	0.413*		0.311
	*p*-value	0.026		0.100
**MMP-9**	Pearson correlation	0.038	0.311	
	*p*-value	0.844	0.100	

* Correlation is significant at the 0.05 level (2-tailed).

** Correlation is significant at the 0.01 level (2-tailed). CPDAI: Composite Psoriatic Disease Activity Index; DAPSA: Disease Activity in Psoriatic Arthritis; MMP-9: Matrix metalloproteinases-9.

**Figure 2.**
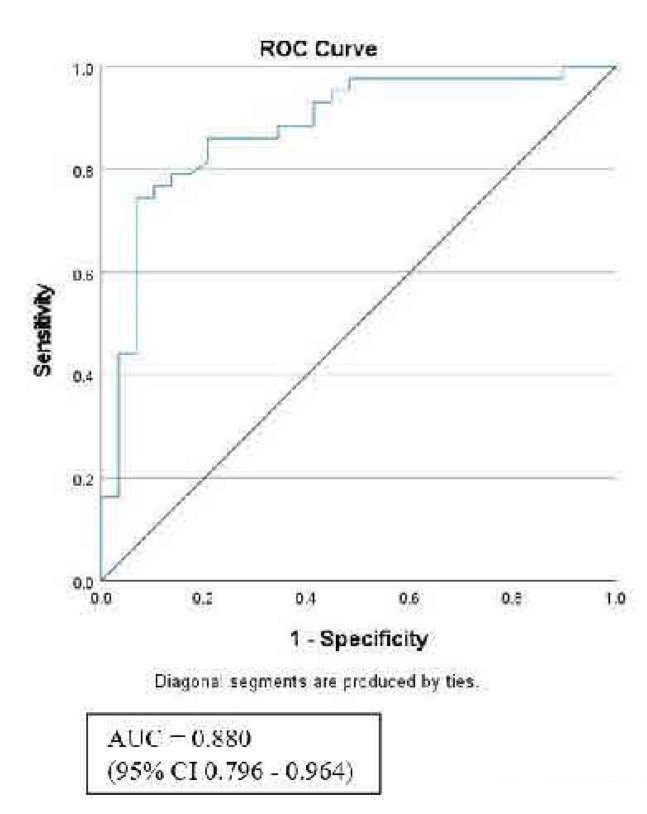
ROC curves showing the AUCs comparing PsA patients (n = 43) with healthy control (n = 29). **AUC = area under the curve; 95% CI = 95% confidence interval.

The area under the receiver operating characteristic (ROC) curve (AUC) provides a measure of the diagnostic accuracy of a biomarker. In this study, the ROC analysis demonstrated that the AUC for MMP-9 is 0.88, which is statistically significant (p < 0.001). This high AUC value indicates that MMP-9 has good diagnostic potential for distinguishing between individuals with PsA patient and healthy controls (**Figure 2**).

## DISCUSSION

PsA is a heterogeneous inflammatory arthritis closely associated with psoriasis, an autoimmune skin condition. In PsA, the inflammation extends to the synovial tissue of the joints, which can lead to joint destruction if left untreated. This ongoing inflammation may result in severe joint damage, causing disability and significantly impacting the quality of life of those affected. The disease manifests in various ways, and its progression can lead to a wide spectrum of symptoms ranging from mild discomfort to severe, disabling arthritis.^13^

This study demonstrates that serum MMP-9 levels are significantly higher in PsA patients compared to the healthy control group, indicating its potential as a diagnostic marker with good accuracy. However, there was no significant correlation found between serum MMP-9 levels and PsA disease activity whether the disease was in the active or inactive state. MMPs are a family of proteolytic enzymes involved in the degradation of extracellular matrix (ECM) components, which is a crucial event in the cartilage destruction and joint erosion seen in inflammatory arthritis like PsA.^14^ Supporting these findings, Fraser et al. showed that synovial fluid MMP-9 levels were significantly higher in early PsA patients compared to those with early rheumatoid arthritis, although the difference was not statistically significant. Additionally, synovial membrane expression of both vascular endothelial growth factor (VEGF) and MMP-9 was elevated in early PsA, but this difference also failed to reach statistical significance. The synovial fluid MMP-9 levels were correlated with the pattern of synovial membrane neovascularisation and synovial fluid VEGF levels in early inflammatory arthritis. Moreover, VEGF has been shown to enhance MMP-9 production by the synovial membrane, suggesting a potential interaction between angiogenesis and MMP activity in the pathogenesis of PsA.^15^

The healthy control group in this study helped confirm the diagnostic power of serum MMP-9 in distinguishing PsA from non-affected individuals. Similarly, a study by Einaz et al. found that the levels of MMP-3 and MMP-9 were significantly elevated in patients with PsA and rheumatoid arthritis compared to those with osteoarthritis.^16^ This suggests that MMP-3 and MMP-9 could be specific markers for joint inflammation and destruction, as they are almost exclusively produced in the inflamed synovium.^17^

Additionally, Jovanovic et al. reported that the production of MMP-9 by monocytes and macrophages is stimulated by inflammatory cytokines such as tumour necrosis factor (TNF), IL-17, which are not specific to RA but are also involved in other diseases with bone erosions such as PsA.^18^ Moreover, Cordiali-Fei et al. demonstrated that therapy with the TNF blocker infliximab in PsA patients resulted in reduced levels of MMP-9, which was associated with an improvement in the Psoriatic Arthritis Severity Index (PASI).^19^ This suggests that TNF blockade not only decreases inflammation but also reduces MMP-9 levels, reinforcing its role in joint damage and disease progression in PsA.

This study reveals that the majority of PsA patients fall into the overweight category, a finding that correlates with a study conducted among PsA patients in Malaysia.^20^ This observation aligns with findings from the US Nurses Health Study II, where BMI and central obesity were associated with an increased risk of developing PsA.^21^ In another study, Anand et al. highlighted that obesity increases the risk of PsA, possibly due to higher levels of pro-inflammatory mediators found in individuals with elevated BMI. Importantly, the author noted that patients with higher BMI are less likely to achieve minimal disease activity (MDA) and are less responsive to therapy.^22^

The limitations of our study should be considered when interpreting the findings, as they pose certain challenges. One of the primary limitations is the small sample size, which may increase the risk of a Type II error, potentially leading to the underestimation of significant associations and affecting the reliability of the results. A larger sample size would be more ideal for yielding statistically robust outcomes and ensuring greater generalisability of the findings. Besides that, this study was conducted during the COVID-19 pandemic, where travel restrictions and the postponement of most patients’ appointments due to the Movement Control Order (MCO) posed additional challenges. This situation may have affected patient recruitment, follow-up schedules, and overall participation in the study, potentially limiting the availability of data and impacting the comprehensiveness of our findings.

Future studies are essential to expand upon the present findings and explore how serum MMP-9 levels compare to other autoimmune diseases involving the joints, such as rheumatoid arthritis, gout, or osteoarthritis. To provide more robust data and guidance to clinical practice, these studies should be conducted prospectively through multi-centred randomised controlled trials with larger sample sizes. Studies that aim to elucidate differences in serum MMP-9 expression between psoriasis and PsA will be crucial. This could help assess the clinical validity and biomarker performance of serum MMP-9 levels. Furthermore, prospective studies should consider longitudinally following psoriasis patients without arthritis to explore whether serum MMP-9, either alone or in combination with other clinical and molecular information, could serve as a predictive marker for developing PsA in those psoriasis patients without evident joint involvement.

This study demonstrated that MMP-9 shows promising potential as a diagnostic marker for PsA. However, while MMP-9 displayed strong diagnostic potential there was no significant correlation between serum MMP-9 levels and PsA disease activity. These findings emphasise the need for further research involving a larger cohort of PsA patients to evaluate whether MMP-9 could play a complementary role in the diagnosis of PsA.

## CONFLICT OF INTEREST

The authors declare no conflicts of interest.

## FUNDING

This research did not receive any specific grant from funding agencies in the public, commercial, or not-for-profit sectors.

## AUTHOR CONTRIBUTIONS

All authors have contributed substantially for the work. All authors have approved the final version to be published, and agreed to be accountable for all aspects of the work in ensuring that questions related to the accuracy or integrity of any part of the work are appropriately investigated and resolved. All authors take full responsibility for the integrity and accuracy of all aspects of the work.

## OPEN DATA SHARING POLICY

The raw data is available with the authors and will be made available on request if needed.
